# Acute psychosis secondary to steroid responsive encephalopathy associated with autoimmune Hashimoto's thyroiditis

**DOI:** 10.1002/ccr3.4644

**Published:** 2021-09-05

**Authors:** Shahd I. Ibrahim, Alaa M. Khalafalla, Abeer S. Safan, Arwa E. Alsaud, Tala B.Batia, Mohamed A. Yassin

**Affiliations:** ^1^ Hamad Medical Corporation Doha Qatar

**Keywords:** encephalopathy, hashimoto thyroiditis, hypothyroid, psychosis, Qatar

## Abstract

Approach to Hashimoto thyroiditis encephalopathy.

## Abstract 2

Approach to Hashimoto thyroiditis encephalopathy

## 1 INTRODUCTION

Hashimoto's thyroiditis (chronic autoimmune thyroiditis) is the most common cause of hypothyroidism in places sufficient iodine supplement. Hashimoto encephalopathy (HE) is a syndrome of acute or subacute encephalopathy that is less reported and most of the times underdiagnosed. Here, we report 36 years old Asian male who presented with features of psychosis and found to have Hashimoto's thyroiditis with elevated thyroid antibodies, who responded very well to pulse steroid therapy in form of 1 gram intravenous methylprednisolone daily for 5 days.

The thyroid is a gland found in the anterior part of the neck which consists of two connected lobes. It mainly secretes three hormones; triiodothyronine (T3), and thyroxine (T4), and a peptide hormone, called calcitonin.^1^, ^2^ Thyroid disorders include hyperthyroidism, hypothyroidism, thyroid inflammation (thyroiditis), thyroid enlargement (goiter), thyroid nodules, and thyroid cancer.^3^

Hypothyroidism is characterized by a decrease secretion of thyroid hormones: the most common cause worldwide is iodine deficiency. Hashimoto's thyroiditis (chronic autoimmune thyroiditis) is the most common cause of hypothyroidism in iodine‐sufficient regions of the world. It occurs due to autoimmune‐mediated destruction in form of diffuse lymphocytic infiltration of the thyroid and follicular destruction which result in gradual failure as well as high serum concentrations of antibodies to thyroid peroxidase (TPO) and thyroglobulin (Tg) in almost all cases.^4^

Hashimoto encephalopathy (HE) is a syndrome of acute or subacute encephalopathy that is associated with elevated antithyroid antibody titers and its diagnosis usually made after exclusion of other causes.

## 2 CASE PRESENTATION

36‐year‐old Asian gentleman with no previous psychiatric or medical illnesses presented to the emergency department with 14 days history of abnormal behavior, in the form of auditory hallucination, delusion of grandeur, and paranoia. He reported hearing the voice of God and said “The Messiah is in the room,” when asked to point, he pointed to his stomach. Collateral history was obtained from his cousin and roommates for which they reported, changes in the patient's baseline behavior 10–14 days prior to his admission, for which he stopped going to work, exhibited aggression and incomprehensible speech about people trying to attack him. There were no reported seizures or abnormal movements, no fever or other constitutional symptoms. He denied any headache and neurological deficits, and his review of systems was unremarkable.

On physical examination, he was vitally stable and afebrile but disoriented to time and place, saying he is in the house of God, he looked bewildered, with dysthymic mood, restricted affect, and tangential thought process. There was no neck stiffness, negative Kernig's and Brudzinski's sign, with normal Cranial nerve and neurological examination, the rest of his physical examination was within normal parameters.

The patient underwent number of investigations shown in Table 1.

**TABLE 1:** Lab valves

Investigation	Patient value on admission	Patient value after 10 days	Patient value upon discharge	Normal Laboratory Value	Unit
TSH	2.62	5.91	3.77	0.3–4.2	mIU/L
Free T3	‒	4.9	6.1	3.7–6.4	pmol/L
Free T4	19.8	25.4	25	11.6–21.9	pmol/L
Anti‐Thyroid Peroxidase	564	‒	‒	0–34	IU/ml
Anti‐Thyroglobulin antibody	19	‒	‒	0–115	IU/ml
Vitamin B12	242			145–596	pmol/L
CSF WBC	0			0–5	
CSF RBC	0			0–2	
CSF glucose	3.92			2.22–3.89	mmol/L
CSF Protein	0.72			0.15–0.45	gm/L

Other investigations including toxicology screen and HIV Antibody/Antigen were negative, radiological investigation such as CT head and MRI/MRA head with contrast showed no abnormality, as well as EEG which was normal.

Hence, a diagnosis of Steroid responsive encephalopathy associated with autoimmune thyroiditis (SREAT) was made. During his hospital course, the neurology team was onboard, and he was started on intravenous pulse methylprednisolone 1 gram daily for five days, followed by 60mg oral prednisolone daily, with a plan of taper down by 10 mg monthly. The patient assessment on daily bases showed significant improvements of his psychotic features on day two of intravenous steroids until it has resolved completely approximately on his tenth day of steroid therapy. The patient then was discharged home with neurology and endocrinology follow‐ups.

## 3 DISCUSSION

Hashimoto's thyroiditis is one of the most common thyroiditis esp. in areas where there is no iodine deficiency, and it is much more common in female than in male.

There many types of Hashimoto's thyroiditis like silent or postpartum forms which tend to be transient. It has been postulated that interaction between genetic and environmental factors leads to development of Hashimoto's thyroiditis in its different forms which includes goitrous and atrophic autoimmune thyroiditis and both have the same pathophysiological and serological features in form of lymphocytic infiltration and high level of thyroid peroxidase (TPO) and thyroglobulin (Tg). ^5^

The disease mainly results in hypothyroid state, however, sometimes the patient may experience thyrotoxicosis symptoms in the beginning of disease due to early inflammatory process and hence thyroid hormone release resulting in what so called “Hashitoxicosis”.^6^

One of the less common presentation of Hashimoto's thyroiditis is Hashimoto encephalopathy (HE) with only 121 cases reported in systematic review published in 2006^7^; however, the disease might be underrecognized as evidenced by one epidemiologic study screened people who presented with neurological symptoms with no known cause and they found that the prevalence may be as high as 0.2%. ^8^

The hallmark of HE is altered mentation in form of confusion, plus or minus accompanying seizures(in approximately two thirds of patients), myoclonus (seen in up to 38% of patients), or loss of consciousness and it has been noticed that 2 patterns of cognitive dysfunction are usually predominant, stroke‐like pattern which is usually more acute to subacute in onset and more gradual diffuse progressive pattern which sometimes results in dementia, hallucination, or even coma in severe cases.

Psychosis, in form of visual hallucinations as well as paranoid delusions, has been reported in 25 to 36 percent of patients [8.9].

The must do investigations in order to diagnose HE in addition to compatible clinical presentation is measurement of antithyroid peroxidase antibody (TPOAb) or antithyroglobulin antibody (TgAb), the finding of elevated level of these antibodies in addition to response to corticosteroids define the syndrome.

The diagnosis of HE is usually made by exclusion, in our case, giving the fact that there was no previous psychiatric history, the rapid onset of the psychosis features, and the rapid response to steroid therapy in addition to presence of antithyroid peroxidase antibody (TPOAb) and antithyroglobulin antibody (TgAb) made the diagnosis of HE as the most likely diagnosis.

Thyroid hormones should be measured as well; however, because their level is usually variable as seen in our case, normal value does not exclude the diagnosis and other possible infectious causes needed to be excluded before starting corticosteroid therapy.^10^

HE is usually treated with corticosteroids and treatment of the underlying thyroid disease. Most patients (90 to 98 percent) respond to steroid therapy. Symptoms typically improve or resolve over a few months. The duration of treatment and the rate of taper are generally titrated to the clinical response. In some patients, this is as long as two years. Several patients have been treated with other immunosuppressive medications, including azathioprine and cyclophosphamide. These are generally reserved for patients who cannot tolerate corticosteroids, or who do not respond to or relapse after or during tapering of corticosteroid therapy.

The mainstay therapy for HE is usually corticosteroids and the response rate is high as up to 90–98%.^9^

Currently, there is no guideline defining the treatment duration and the rate of tapering of corticosteroid and its usually guided by clinical response^10^ and in some cases other immunosuppressive medications such as azathioprine and cyclophosphamide in cases of corticosteroids intolerance, failure to respond, or relapse.^11^

Seizures as one of the presentations of HE can be managed by phenytoin; however, in some case, there was no response to anticonvulsant medications and the patient responded well to steroid therapy.^12^

The prognosis of HE is generally good and spontaneous recovery has been observed even in cases where there was delay in the diagnosis up to few years as suggested by some case series, however, there around 25 percent possibility of having residual cognitive impairment in such cases.^12^, ^13^

One series of 20 patients has reported around 15 percent rate of relapse after steroids discontinuation.^13^

## CONFLICT OF INTEREST

None.

## AUTHOR CONTRIBUTIONS

Shahd I. Ibrahim and Mohamed A. Yassin: Manuscript writing. Alaa M. Khalafalla, Tala B. Batia, and Arwa E. Alsaud: Literature review. Abeer S. Safan: Primary treating physician and literature review. Authorship: All authors had access to the data and played a role in writing the manuscript.

## ETHICAL APPROVAL

This case report has granted ethical approval by Medical Research Center—Hamad medical corporation, Qatar.
